# Global transcriptional analysis reveals surface remodeling of *Anaplasma marginale* in the tick vector

**DOI:** 10.1186/1756-3305-7-193

**Published:** 2014-04-21

**Authors:** G Kenitra Hammac, Sebastián Aguilar Pierlé, Xiaoya Cheng, Glen A Scoles, Kelly A Brayton

**Affiliations:** 1 Program in Genomics, Department of Veterinary Microbiology and Pathology, Paul G. Allen School for Global Animal Health, Washington State University, Pullman, WA 99164-7040, USA; 2 Animal Disease Research Unit, U.S. Department of Agriculture, Agricultural Research Service, PO Box 646630, Pullman, WA 99164-6630, USA; 3 Present address: Department of Comparative Pathobiology, Purdue University, West Lafayette, IN 47907, USA

**Keywords:** Global transcription, Intracellular bacteria, RNA-seq, Transcriptomics, Surface proteins

## Abstract

**Background:**

Pathogens dependent upon vectors for transmission to new hosts undergo environment specific changes in gene transcription dependent on whether they are replicating in the vector or the mammalian host. Differential gene transcription, especially of potential vaccine candidates, is of interest in *Anaplasma marginale*, the tick-borne causative agent of bovine anaplasmosis.

**Methods:**

RNA-seq technology allowed a comprehensive analysis of the transcriptional status of *A. marginale* genes in two conditions: bovine host blood and tick derived cell culture, a model for the tick vector. Quantitative PCR was used to assess transcription of a set of genes in *A. marginale* infected tick midguts and salivary glands at two time points during the transmission cycle.

**Results:**

Genes belonging to fourteen pathways or component groups were found to be differentially transcribed in *A. marginale* in the bovine host versus the tick vector. One of the most significantly altered groups was composed of surface proteins. Of the 56 genes included in the surface protein group, eight were up regulated and 26 were down regulated. The down regulated surface protein encoding genes include several that are well studied due to their immunogenicity and function. Quantitative PCR of a set of genes demonstrated that transcription in tick cell culture most closely approximates transcription in salivary glands of recently infected ticks.

**Conclusions:**

The ISE6 tick cell culture line is an acceptable model for early infection in tick salivary glands, and reveals disproportionate down regulation of surface protein genes in the tick. Transcriptional profiling in other cell lines may help us simulate additional microenvironments. Understanding vector-specific alteration of gene transcription, especially of surface protein encoding genes, may aid in the development of vaccines or transmission blocking therapies.

## Background

Pathogens dependent upon vectors for transmission to new hosts undergo environment specific changes in gene transcription dependent on whether they are replicating in the vector or the vertebrate host. This dynamic relationship has been investigated in several human pathogens. For example, transcriptional analysis of *Ehrlichia chaffeensis*, the causative agent of human monocytic ehrlichiosis, revealed increased transcriptional activity and expression levels in tick cells versus human cells [1]. There is an inverse relationship between the transcription levels of outer surface proteins (OSP) A and C of *Borrelia burgdorferi*, the causative agent of Lyme disease, indicative of mammalian host and vector specific protein expression [2,3]. Similarly, differential transcription of *Anaplasma phagocytophilum* genes between tick and human cell lines was disproportionately represented by membrane or surface proteins [4]. Vector-specific alteration of gene transcription sheds light onto the mechanisms that orchestrate the transition between mammalian host and vector, which is especially of interest in the context of surface protein-encoding genes as these molecules are potential targets for vaccine development or transmission blocking therapies.

We are interested in exploring differences in pathogen transcription in these dissimilar environments and are using *Anaplasma marginale* as our model. *A. marginale* is an obligate intracellular pathogen that causes bovine anaplasmosis and depends on a tick vector for efficient transmission. In the bovine host *A. marginale* infects the erythrocyte and replicates up to levels of 10^9^ bacteria per ml of blood. When ticks feed on infected cattle, *A. marginale* first invades and colonizes the midgut epithelium in a receptor mediated process involving the surface protein Msp1a [5-9]. Within the midgut epithelial cells, *A. marginale* replicates within intracellular vacuoles to form colonies of up to several hundred organisms per cell. After this initial replication in the midgut epithelium, *A. marginale* enters the hemolymph and subsequently invades the salivary gland epithelial cells [6,10,11]. Final replication of up to 10^6^ organisms per salivary gland pair and development of infectivity for cattle requires re-attachment and initiation of feeding followed by inoculation of *A. marginale* with the saliva into the susceptible host [10-12]. Thus, *A. marginale* transmission requires that it efficiently invades and replicates in tick tissues, culminating in the development of infectivity in the salivary gland. Because of the relevance of the pathogen-vector transition, much work has gone into examining changes the pathogen undergoes in these two different environments.

Because of technical challenges associated with tick rearing, infection and processing, cultured tick cells have been used as a model for the tick phase of the *A. marginale* life cycle. Specifically, continuous embryonic cell lines from *Ixodes scapularis* (ISE6 or IDE8) were used in this and previous studies [13,14]. Altered gene transcription and expression in *A. marginale* has been demonstrated between bovine erythrocytes and tissues of the tick vector or cultured tick cells by quantitative PCR, immunoblotting and comparative mass spectrometric analysis [15-19]. In addition to quantitative change, more structurally complex variants of *msp2* have been shown to predominate in mammalian versus tick cell lines suggesting a functional purpose for the complexity of the variants [20].

High throughput sequencing technologies used to obtain information about a sample’s RNA content, or RNA-seq, have proven to be reliable tools for determining whole genome transcriptional activity in many species including obligate intracellular bacteria [21-23]. In the present study, the power of RNA-seq was harnessed to study the transcriptional dynamics of *A. marginale* in tick cell culture and bovine erythrocytes. The power and reproducibility of this technique allowed for a more comprehensive analysis than has been possible with other methods. Comparison of transcript levels from organisms isolated from these two environments reveals modulation of 14 pathways or component groups, with surface protein genes showing the highest degree of modulation in the St. Maries strain of *A. marginale* between the bovine host and tick cell culture. These observations allowed us to test whether tick cell culture is a suitable model for the tick vector.

## Methods

### Ethics statement

Animal experiments were approved by the Institutional Animal Care and Use Committee at Washington State University, USA, in accordance with institutional guidelines based on the U.S. National Institutes of Health (NIH) Guide for the Care and Use of Laboratory Animals (ASAF 4440).

### Deep sequencing

RNA was prepared from two biological replicates of *A. marginale* St. Maries strain maintained in ISE6 cell culture as previously described [13,14,24] and two biological replicates from infected bovine blood [21]. Samples were collected and processed using TRIzol (Invitrogen, Carlsbad, CA, USA) for RNA extraction, the MICROBEnrich kit (Ambion, Austin, TX, USA) to negatively select eukaryotic RNA and were normalized using Duplex-Specific thermostable Nuclease (DSN) (Illumina, San Diego, CA, USA) [21,25]. Samples were sequenced with Illumina technology with 100 base pair reads [25]. The accession numbers for this RNA-seq study in the GenBank Sequence Read Archive (SRA) are SRP014580 and SRP014580.

### Comparative transcriptional analysis

CLC Genomics Workbench 6.0.2 (CLC Bio, Cambridge, MA, USA) was used to process RNA-seq data. Expression levels of genes were normalized by both library size and gene length effects by use of the reads per kilobase of gene model per million mapped reads (RPKM) [26]. Transcriptional fold change was determined between two conditions: *A. marginale* in ISE6 cell culture and blood. For inclusion in the final analysis, normalized fold change calculations had a p-value of <0.01 according to Kal’s Z-test [27]. Fold changes were considered significant for surface proteins if greater than two standard deviations above or below the average fold change across all genes between replicates. Briefly, when differential expression is defined as greater than two standard deviations from the mean, genes with fold changes greater or smaller than the calculated value can be classified as up- or down-regulated at a confidence level of approximately 95% [28]. To determine if surface protein transcription differed between conditions as a group, a category was created including all surface exposed proteins encoded by a single gene and was compared to previously established categories by gene set enrichment analysis (GSEA) [25,29]. Significantly altered categories or pathways were considered at p < 0.05.

### K-means clustering

The mean transcriptional value of each gene to the cluster whose center is nearest was assigned using K-means clustering. This procedure was applied to Illumina data in order to assess the general behavior of the putative protein surface encoding genes. Lloyd’s algorithm was used for these experiments [30]. Euclidean distance was used as distance metric; four partitions were used to generate the clusters. For each gene, the mean gene expression value over all input samples was subtracted. Normalized expression values were used for clustering. Once gene clusters were generated, seven surface encoding genes present in Cluster 3 and three genes present in Cluster 1 were chosen for qRT-PCR validation of Illumina data. Silhouette Indices [31] were calculated for these genes to verify that they accurately represent their Cluster and its transcriptional behavior, and therefore are adequate for RNA-seq data validation.

### Tick samples

Male *Dermacentor andersoni* ticks of the Reynolds Creek stock that have been demonstrated to efficiently transmit *A. marginale* St. Maries were used in this experiment [32,33]. Naïve 5 month old calf 40420 was injected intravenously with *A. marginale* St. Maries stabilate, and ticks were applied 26 days later when the percent parasitized erythrocytes (PPE) was 0.94%. Ticks acquisition fed for seven days, including during the peak of infection at 3.4% on day 28, then were gently removed. Ticks were held at 26˚C in 97% humidity and 12 hours light: 12 hours dark photoperiod for seven days to allow clearance of the blood meal from the mouthparts in order to prevent mechanical transmission of *A. marginale*. Subsequently, ticks were applied to naïve 5 month old calf 41431 for a seven day transmission feed. Both calves were determined to be negative for antibodies to *A. marginale* by competitive ELISA (VMRD, Pullman, WA, USA) prior to experimental infection [34]. Cohorts of post-acquisition ticks and post-transmission ticks were dissected after a three day hold at 26˚C in 97% humidity and 12 hours light: 12 hours dark photoperiod. Individual salivary gland pairs and midguts were collected and stored in cell lysis buffer (Gentra/Qiagen, Valencia, CA, USA) with 200 μg/mL proteinase K for DNA extraction, and pools of 10 salivary gland pairs or midguts were stored in 200 μL RNA Later (Qiagen) for RNA extraction [15]. Genomic DNA was extracted from individual tick salivary gland pairs and midguts following an overnight incubation at 56˚C as previously described [11]. To determine pathogen load, qRT-PCR was performed using primers specific for the single copy gene *msp5*[35]. RNA was extracted from tick salivary glands and midguts using Qiashredder and RNeasy (Qiagen), and then treated with TurboDNAse (Ambion) as previously described [36].

### Quantitative PCR

qRT-PCR was used to confirm RNA-seq results from blood and cell culture samples and to determine transcription levels in tick samples. Complementary DNA was synthesized from total RNA using Vilo Superscript (Invitrogen). Ten genes differentially regulated according to the RNA-seq data were targeted with qRT-PCR (SybrGreen, Invitrogen), in addition to the constitutively expressed *msp5* used for normalization [15,25]. Primers used in this study are shown in Table 1. The delta delta Ct (ΔΔCt) calculation was employed to calculate relative change [37]. Bootstrap analysis assigned significance to the fold change values based on consistency among replicates [38].

**Table 1:** Oligonucleotides for qPCR

**Name**	**Sequence (5′-3′)**	**Target gene**
*AM008 F*	ATGCAGCAGCGTGGAGAAGCT	*AM008*
*AM008 R*	AAGCTGGTCCTGTGTGGTTGTTACGT	
*AM009 F*	AAGGGACGGCGAAGTCACAGC	*AM009*
*AM009 R*	TACTTGGACCTCAGGGTACATTTGGCT	
*AM360 F*	TTGACTTACTCGCTGGTATCGCCTACAA	*AM360*
*AM360 R*	AAGAGTACCCAAGTATGCCAAAACCCGA	
*AM366 F*	TATGGCGAGGAAGGCGTTCAAAGTC	*AM366*
*AM366 R*	TGCGAAGCACCGTACATGACGATT	
*AM779 F*	AGGACCACAACCCCATCATGTTTGT	*AM779*
*AM779 R*	TTCCCTAGAGCAGAGGTCTAGTGAGT	
*Msp3 F1 (2281)*	AACCCAACTTTCAACGGTATCAAGGACCT	*msp3*
*Msp3 R1 (2528)*	ATCCCTACTTCAACCCTGGCTCCT	
*Msp1b F (229)*	TACGAGAGCGTGGGACTACGTGCTA	*msp1b*
*Msp1b R (439)*	AAGCTGCTGCCTTGCCAAATTCTTG	
*New msp5 F*	AAGTTGTAAGTGAGGGCATAGCCTCC	*msp5*
*New msp5 R*	AACTTATCGGCATGGTCGCCTAGT	
*Omp4 F*	TTCCAACACACAGGAGGTGACACAC	*omp4*
*Omp4 R*	TTCTCTGCACCATAGCCCGCAA	
*Omp8 F*	TTGCCCGAGCACCGAGATTTCT	*omp8*
*Omp8 R*	ATGGCTTTGCGTCTCCGTTCAG	
*virB10 F*	ATCGTGGACGTAAGGACATTCCCCA	*virB10*
*virB10 R*	TGACTGTGAGTTGGTCTAGGGTCATCC	

## Results

### Transcriptional analysis of surface protein genes

**Figure 1:**
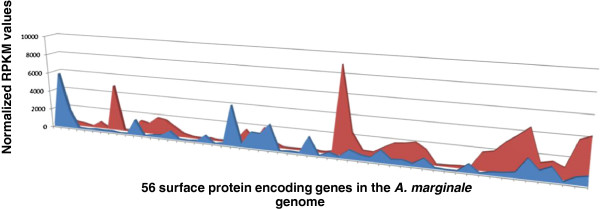
**Whole genome comparison of*A. marginale*transcriptional activity in ISE6 cells v. blood.** The normalized RPKM values for 56 surface protein encoding genes are shown on the y axis. Features are arranged from left to right as they appear on the *A. marginale* chromosome on the x axis. RPKM values for *A. marginale* transcription in ISE6 cells are shown in blue; values for transcripts in blood are shown in red.

RNA from *A. marginale* in tick cell culture and bovine erythrocytes was analyzed by RNA-seq. Analysis of global transcriptional patterns showed that several of the most down regulated genes in tick cell culture corresponded to those that encoded surface proteins. Therefore, we examined genes encoding surface proteins for their transcriptional status. *A. marginale* has a relatively small number of known surface proteins, thus, only fifty-six single copy genes were included in the analysis based on genomic and proteomic evidence that they encode surface proteins [39]. Proteins encoded by more than one gene were eliminated from the analysis due to ambiguous mapping of sequenced reads. Fold changes were considered significant if greater than two standard deviations above or below the average fold change across all surface genes between replicates, and with a p-value below 0.01. Based on these criteria, 26 surface protein encoding genes had decreased transcription in ISE6 culture, and eight surface protein encoding genes had increased transcription (Figure 1, Table 2, Additional file 1). Analysis of relative transcription of all *A. marginale* genes in ISE6 culture compared to bovine blood indicated a similar percentage of up and down regulated genes (20.5% and 18.6%, respectively), whereas genes encoding surface proteins were generally transcribed at a lower level in tick cells (46.4%) (Table 3).

**Table 2:** Genes encoding surface proteins differentially transcribed in ISE6 culture v. blood

**Genome ID**	**Functional annotation**	**Fold change ISE6/Blood**	**ISE6 RPKM**	**Blood RPKM**
AM778	Outer membrane protein	9.8	4221.0	430.2
AM090	MSP4	6.7	5950.9	891.8
AM956	Cytosol aminopeptidase; pepA	6.6	1827.0	275.6
AM854	Putative peptidoglycan-associated lipoprotein	5.0	2672.7	532.8
AM097	VirB9-1	4.4	1925.6	434.9
AM197	Undefined product	3.9	54.9	14.2
AM1166	OMP5	3.8	828.7	216.5
AM560	Putative cell-surface protein	3.5	787.4	226.7
AM1142	OpAG2	-3.5	516.0	1827.0
AM123	MSP1b2	-3.6	90.7	330.6
AM1140	OpAG3	-4.0	434.0	1727.4
AM573	Paralog of AM560	-4.2	13.5	56.5
AM878	AAAP	-4.4	21.6	95.9
AM1315	VirB9-2	-4.7	898.2	4221.0
AM779	Outer membrane protein	-4.8	317.5	1533.7
AM532	MLP2	-4.9	78.7	386.4
AM535	MLP3	-5.1	31.6	162.4
AM127	Undefined product	-5.2	134.1	692.9
AM1159	OMP3	-5.4	23.9	128.6
AM1314	virB10	-5.4	698.9	3795.2
AM529	Undefined product	-5.5	301.3	1648.1
AM1164	OMP4	-5.5	37.9	207.7
AM987	OMP15	-5.6	14.2	79.4
AM1220	OMP7	-6.3	309.9	1960.9
AM188	Partial gene of msp1b gene family; msp1Bpg1	-6.5	7.3	47.0
AM1222	OMP9	-7.0	532.2	3703.0
AM1258	OMP13	-7.1	155.7	1110.2
AM1143	OpAG1	-7.6	268.9	2040.0
AM1221	OMP8	-7.8	377.6	2929.1
AM387	Undefined product	-8.6	210.2	1809.7
AM1219	OMP6	-22.1	79.4	1753.8
AM180	MSP1b1	-35.1	138.4	4857.2
AM366	Undefined product	-46.9	23.2	1085.6
AM1063	MSP3	-55.2	162.8	8984.4

**Table 3:** **Percentages of** ***A. marginale*** **differentially transcribed genes in ISE6 culture v. bovine blood**

	**All genes (952)**	**Surface protein-encoding genes (56)**
Increased	195 (20.5%)	8 (14.3%)
Decreased	177 (18.6%)	26 (46.4%)
Unchanged	580 (60.9%)	22 (39.3%)

Gene set enrichment analysis (GSEA) and custom annotation files allowed for analysis of all genes encoding surface proteins as a group. This group of genes was evaluated in the context of the other known gene categories and pathways in the *A. marginale* genome [25]. GSEA identified 14 significantly altered gene categories between *A. marginale* isolated from bovine blood and tick cell culture. The groups most significantly different (p < 0.01) between the studied conditions included members of the Type IV Secretion System (T4SS), the pathogenesis category, and surface proteins. The latter category displayed the lowest p-value (Table 4).

**Table 4:** **Significantly differentially transcribed** ***A. marginale*** **genes when comparing ISE6 v. blood with GSEA**

**Category**	**Description**	**Test statistic**	**Lower tail**^**a**^	**Upper tail**^**a**^
**19867**	Outer membrane	-675.412	0^b^	1
**30255**	Protein secretion by the type IV secretion system (PMID:11895979 [TAS])	-423.133	0^b^	1
**9405**	Pathogenesis (GO_REF:0000011 [ISS] TIGR_TIGRFAMS:TIGR02800)	-131.569	0.0046	0.9954
**6289**	Nucleotide-excision repair (PMID:16482227 [ISS] TIGR_TIGRFAMS:TIGR00630)	-86.7752	0.0155	0.9845
**8152**	Metabolic process (PMID:16482227 [ISS] UniProtKB:P28304)	-49.7659	0.0208	0.9792
**9253**	Peptidoglycan catabolic process (PMID:10952301 [ISS])	-38.009	0.0273	0.9727
**6810**	Transport (PMID:12704232 [ISS] Pfam:PF02472)	-48.3038	0.0421	0.9579
**6298**	Mismatch repair (PMID:16482227 [ISS] TIGR_TIGRFAMS:TIGR01070)	-24.2468	0.0476	0.9524
**9089**	Lysine biosynthetic process via diaminopimelate (GO_REF:0000011 [ISS] TIGR_TIGRFAMS:TIGR00656)	62.61262	0.9557	0.0443
**6556**	S-adenosylmethionine biosynthetic process (PMID:16482227 [ISS] TIGR_TIGRFAMS:TIGR01034)	51.23363	0.957	0.043
**6436**	Tryptophanyl-tRNA aminoacylation (PMID:16482227 [ISS] TIGR_TIGRFAMS:TIGR00233)	70.56015	0.9705	0.0295
**16114**	Terpenoid biosynthetic process (PMID:16482227 [ISS] TIGR_TIGRFAMS:TIGR00154)	60.77005	0.9706	0.0294
**42255**	Ribosome assembly (PMID:16482227 [ISS] Pfam:PF00573)	1.8E + 308	0.9757	0.0243
**6402**	mRNA catabolic process (PMID:16482227 [ISS] UniProtKB:P05055)	82.09836	0.9873	0.0127

^a^Lower/Upper tail: Lower and Upper tail values show the mass in the permutation based p-value distribution below or above the value of the test statistic.

^b^P values represented as 0 are less than 10^-16^.

**Figure 2:**
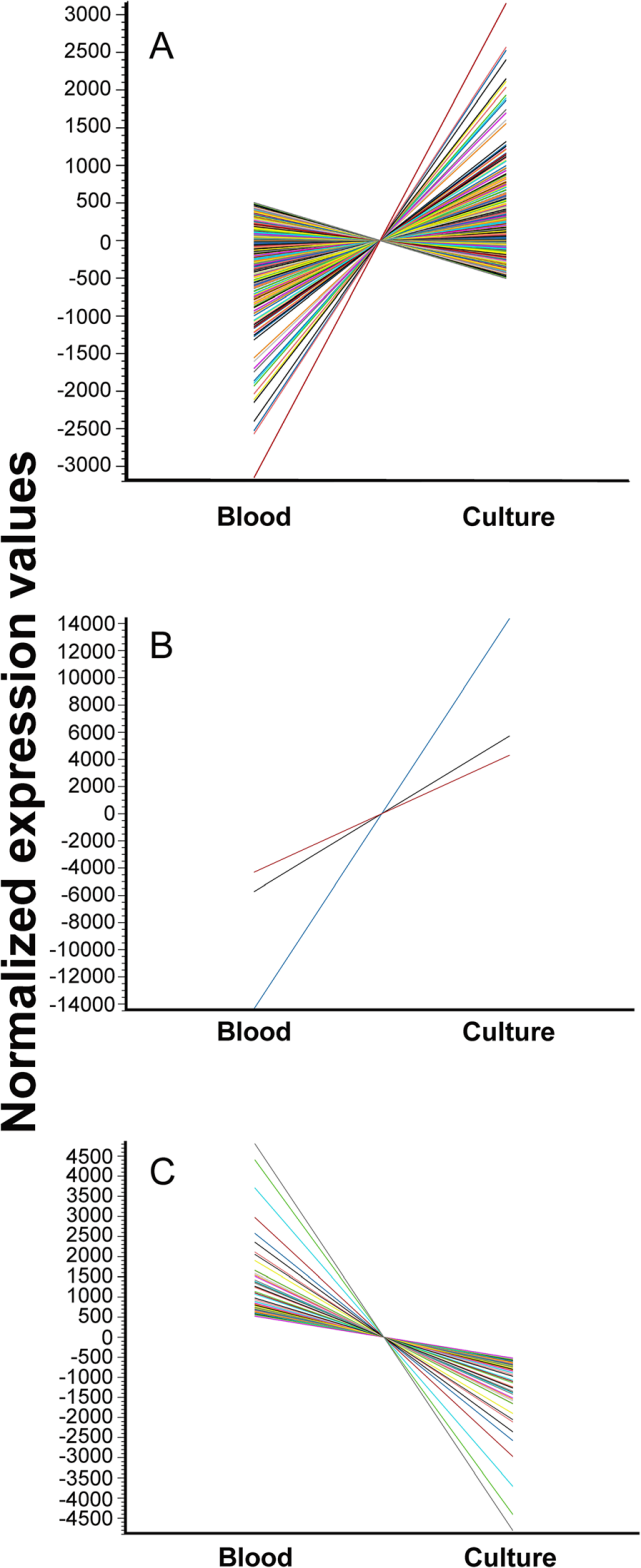
**Gene clusters representing differential transcription in ISE6 cells v. blood.** The normalized expression values for the genes included in Clusters 2 **(A)**, 1 **(B)** and 3 **(C)** as defined by K-means clustering are shown on the y axis. The x axis represents the two conditions in which the *A. marginale* were grown, bovine blood and tick cell culture. Each gene is represented by a line of unique color. Cluster 4, with a single gene down regulated in cell culture is not shown.

K-means clustering was used to confirm trends followed by surface protein encoding genes and to identify genes for qRT-PCR validation of RNA-seq data. Most genes (874) fell into Cluster 2, which is comprised of genes that were mostly up regulated and slightly down regulated between blood and tick cell culture conditions. Clusters 1 and 4 grouped a small number of genes, only three and one respectively. This is not surprising as they cluster the most dramatically up- and down-regulated genes. The difference in average expression values for these clusters was much greater than the differences seen in Cluster 2. Cluster 3 was of special interest as it included a total of 78 genes that were significantly down regulated in cell culture, including 15 of the surface protein encoding genes. Seven of the genes from Cluster 3 and all three genes from Cluster 1 were chosen to validate the RNA-seq data (Figure 2). Silhouette indexes were calculated for these genes in order to corroborate proximity to their own clusters. The silhouette index confirmed the trend for 9 out of 10 genes, with *omp4* not being validated by the analysis. The confidence indicator calculated for *omp4* indicated that it actually belonged to Cluster 2.

### Validation of RNA-seq results by qRT-PCR

Quantitative real-time PCR (qRT-PCR) was performed on a set of ten genes differentially regulated according to the RNA-seq data (*am008, am009, am360, am366, am779, msp1B1*, *msp3*, *omp4*, *omp8, virb10*), and the constitutively expressed *msp5* was used for normalization. Differential regulation shown by RNA-seq was confirmed by qRT-PCR for eight out of the ten genes examined (Table 5). The two genes whose trends were not confirmed, *am366* and *am779*, are annotated as hypothetical and an outer membrane protein, respectively. They were down regulated in ISE6 culture according to RNA-seq analysis, but up regulated according to qRT-PCR.

**Table 5:** qPCR transcriptional fold change determination in ISE6 and tick samples v. blood

	**RNA-seq **			**qPCR **		
	**ISE6 v Blood**	**ISE6 v Blood**	**SGpa v Blood**	**SGpt v Blood**	**MGpa v Blood**	**MGpt v Blood**
am366	-46.9	6.6	1.1	18.5	10.9	-4.4
am779	-4.8	2.6	-2.3	-1.3	1.1	1.7
msp1b	-35.1	-8.3	-5.9	1.5	-9.6	-1.2
msp3	-55.2	-4.5	-15.0	1.4	1.1	-7.3
omp4	-5.5	-7.1	-3.8	-53.7	1.2	3.3
omp8	-7.8	-1.8	-1.4	1.8	2.2	2.9
virb10	-5.4	-6.9	-16.0	-4.9	-1.3	-4.1
am008	61.0	14.4	138.8	25.5	19.4	78.1
am009	303.0	10.1	8.0	26.1	8.5	127.4
am360	8655.0	34.6	13.1	1.9	84.2	4.2

### qRT-PCR of *A. marginale* RNA from tick salivary glands and midguts

**Figure 3:**
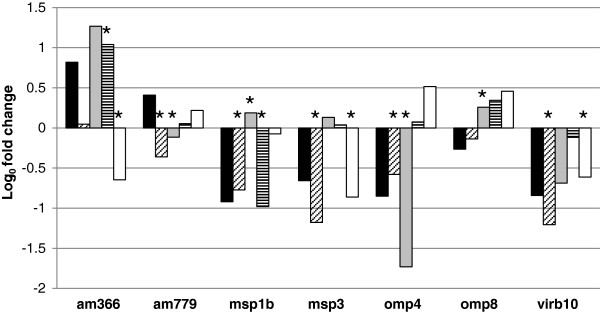
**qPCR transcriptional fold change determination in ISE6 and tick samples v. blood.** Graphical representation of seven cell surface protein encoding genes’ transcriptional fold change in tick samples versus bovine blood as determined by qRT-PCR. ISE6 is represented by black bars, SGpa by bars containing diagonal stripes, SGpt by gray bars, MGpa by bars containing horizontal stripes and MGpt by white bars. Bootstrap analysis assigned significance (*) to the fold change values based on consistency among replicates.

In order to collect tissues representing a complete transmission cycle ticks were fed on an *A. marginale* infected calf to acquire the organism and then allowed to feed on a naïve calf to transmit the organism following typical feeding and holding times. These ticks were confirmed to be positive for *A. marginale*, with 100% infection rates and a mean infection level of 10^5^ organisms per salivary gland pair and midgut by qRT-PCR targeting *msp5* (data not shown). RNA was extracted from salivary glands and midguts from cohorts of post-acquisition (pa) and post-transmission (pt) fed ticks. qRT-PCR targeting seven down regulated and three up regulated genes revealed surface protein gene transcription of *A. marginale* in tick cell culture mimics early infection in the tick salivary gland (Figure 3, Table 5). The three up regulated genes, according to RNA-seq, included in this analysis (*am008*, *am009*, *am360*) were confirmed as up regulated in all tick conditions: salivary glands post-acquisition (SGpa), salivary glands post-transmission (SGpt), midguts post-acquisition (MGpa), and tick midguts post-transmission (MGpt). The tick condition most similar to ISE6 culture was the post-acquisition salivary glands in which five of the seven *A. marginale* genes tested were down regulated in SGpa in addition to ISE6 as determined by both RNA-seq and qRT-PCR. Consistent with RNA-seq data, *am779* was down regulated in SGpa, while *am366* was slightly up regulated, consistent with qRT-PCR data comparing ISE6 versus blood. The only gene consistently down regulated in all tick conditions was *virB10*. Genes down regulated in ISE6 cells according to RNA-seq were less likely to be down regulated in the following tick conditions: SGpt, MGpa and MGpt. Aside from SGpa, transcription in other tick samples was dissimilar to that in ISE6 cells, indicating differences in environmental conditions may result in altered gene transcription.

## Discussion

RNA-seq data revealed a disproportionate down regulation of surface protein gene transcription in tick cell culture compared to bovine blood. Additionally, GSEA analysis found the group of surface protein encoding genes to be altered, warranting a more in depth analysis of these genes. Relatively little is known about the eight surface protein encoding genes up regulated in tick cell culture, whereas the 26 down regulated surface protein encoding genes include several well-studied genes due to their immunogenicity and function. The steady-state gene, *msp5*, was confirmed by RNA-seq not to be differentially transcribed between the two studied conditions; the protein product for this gene has also been demonstrated to be steady state [17]. Complete sequencing of the St. Maries strain of *A. marginale* revealed that over three quarters of surface proteins belong to two superfamilies (the *msp2* and *msp1* superfamilies), both of which were found to be down regulated in tick cell culture in this study [40].

Members of the *msp2* superfamily were generally transcribed at a lower level in tick cell culture, with the exception of *msp2*, *msp4* and *omp5*. Lower levels of transcription in ISE6 cells were observed for several *omps, opag3* and *msp3*, which had the lowest relative transcription of all surface proteins in *A. marginale* infected cell culture. Down regulation of *msp3* was confirmed by qRT-PCR, but the fold change was not as great as determined by RNA-seq. This can be explained by specificity of qRT-PCR primers for a sequence specific to the single *msp3* expression site, while the RNA-seq data were skewed by sequence reads from expression site variants that were not represented in the sequence used for mapping (i.e. a single *msp3* sequence is represented in the expression site of the genome sequence, while the RNA-seq data were obtained from a population of organisms expressing different *msp3* variants). Transcription of the immunodominant and antigenically variable *msp2* was not significantly different between conditions, while all other members of the same operon (*opags 1, 2, 3*) were significantly down regulated in culture. It is not clear why *msp2* would behave differently from other members of its operon, but it is likely that *msp2* has lower transcription in tick cell culture consistent with other members of its operon and the *msp2* homolog in *A. phagocytophilum* (*p44*) [18,40,41]. One explanation hinges on *msp2* variants differing between the bovine host and the arthropod vector [20]. Msp2 variants expressed in the tick vector are known to be less complex than variants expressed in the bovine host, as they have been shown to incorporate segments derived from fewer donor pseudogene sequences than variants identified in blood. The lower complexity of tick expressed variants would facilitate mapping to the “simple” *msp2* expression site contained in the genome sequence. It appears that the reads from *A. marginale* infected ISE6 culture were mapped more efficiently to the *msp2* expression site in the genome sequence than the reads from blood, resulting in enhancement of the reads from culture and diminution of the reads from blood. Down regulation of *msp2* transcription in ISE6 culture may have been masked, and thus seen as an insignificant fold change between conditions.

Nine of the 15 genes encoding “outer membrane proteins” (OMPs) of the *msp2* superfamily were differentially transcribed, with eight having lower transcription levels in ISE6 cells compared to bovine blood. Relative transcription and expression of OMPs in bovine erythrocytes and a different tick cell line (IDE8) or tick MGs and SGs have previously been examined by immunoblotting and qRT-PCR [16]. Of the seven OMPs previously demonstrated to be down regulated in IDE8 tick cell culture, RNA-seq confirmed lower transcription levels in tick cell culture of four: *omp 4, 7, 8* and *9*. The remaining three previously shown to be down regulated (*omp1*, *10* and *11*) were not differentially transcribed between ISE6 culture and bovine erythrocytes in the present experiment. Lower relative protein expression in IDE8 cells was also demonstrated in the previous study for *omp 1, 4, 7, 8, 9* and *11*, while *omp 10* was undetectable in both blood and IDE8 cells by immunoblotting [16]. *A. phagocytophilum omp1* has been shown to be down regulated in ISE6 culture compared to a human cell line [4]. Disagreement between RNA-seq and previous studies may reside in inherent differences in techniques, cell lines used, their infection levels and interpretation criteria. Also, transcription levels of orthologs in *A. phagocytophilum* may not be a good indicator of transcription in *A. marginale* as these species have different tick vectors and vertebrate host cell tropism.

The smaller *msp1* superfamily was also generally down regulated in *A. marginale* infected tick cell culture as compared to bovine erythrocytes. Four members of this six gene family were transcribed at significantly lower levels in tick cells (*msp1b1*, *msp1b2*, *mlp2*, *mlp3*), and the other two members had comparable levels of transcription in the erythrocytes versus tick cells (*msp1a* and *mlp4*). These data were in contrast to previously published data that *msp1b* was transcribed and expressed at similar levels in blood and tick cells and *msp1a* had decreased transcription and expression in tick cells compared to blood [42]. The finding that *msp1b1* and *msp1b2* are down regulated in tick cells makes sense in the context of previously demonstrated adhesion properties of *msp1b* in bovine erythrocytes, but not in tick cells [43]. Additionally, *msp1a* which has adhesin properties in both bovine erythrocytes and tick cells was not differentially transcribed [43].

Two members of the T4SS, *virB9-2* and *virB10*, were transcribed at a lower level in tick cell culture, while a third T4SS member, *virB9*-1, was up regulated. The T4SS has a role in effector translocation, and *virB9-1*, *virB9-2* and *virB10* are components of the core T4SS complex that forms a secretory channel between the bacterium’s inner and outer membranes [44]. Immunoprecipitation studies have shown that VirB9-2 and VirB10 both interact with VirB9-1, but not each other, suggesting that VirB9-1 is a central part of the structure forming the core complex of the T4SS [45]. Down and up regulation of *virB9-2* and *virB9-1* are of approximately equal magnitude, -4.7 and 4.4 fold change, respectively. Alternate transcription of *virB9-1* and *virB9-2* in ticks versus the host may indicate a functional change in the T4SS in the two environments. The trend of down regulation was confirmed for *virB10* in all tick samples studied, indicating consistent down regulation in tick tissues through all stages of infection. GSEA further confirmed this observation as the category composed of T4SS genes was one of the most significantly altered. Because a specific antibody response is mounted against *virB9-1*, *virB9-2* and *virB10* in outer membrane-vaccinated and naturally infected calves, they have been targeted in vaccine studies which showed broad MCH class II presentation and interactions which enhance their immunogenicity if used as a linked protein vaccine [45-48]. In contrast to the findings of this study, tiling arrays have previously shown increased transcription of *A. phagocytophilum* T4SS genes in ISE6 culture as compared to mammalian cell lines, once again indicating that *A. phagocytophilum* transcription is a poor indicator of transcription in *A. marginale*[4].

The *Anaplasma* appendage-associated protein (AAAP) was also transcribed at a lower level in *A. marginale* infected culture compared to blood. A role for AAAP in both blood and ticks is expected as F-actin appendages are assembled on the cytoplasmic surface of *A. marginale* containing vacuoles within erythrocytes and appendages are associated with *A. marginale* in the tick blood meal and tick midgut [49,50]. Variation in expression levels between strains has been demonstrated, so it is possible that our finding is strain-specific [49].

Previous characterization of the *A. marginale* proteome between the bovine host and vector identified AM778 to be expressed only in the tick [17,51]. Consistent with previous studies, RNA-seq found *am778* to be the most highly up regulated surface protein in ISE6 cells. Distantly related paralogs, AM779 and AM780, have been identified in protein complexes from *A. marginale* in blood, but not in ISE6 culture [51]. RNA-seq supported this previous observation by showing *am779* and *am780* to be down regulated in ISE6 culture, however, the fold change of *am780* was not considered significant by the parameters set for this study. Down regulation of *am779* in ISE6 cells was not confirmed by qRT-PCR. AM779 has been examined as a subdominant antigen, but did not provide protective immunity against challenge in immunized animals [52]. The RNA-seq data suggests alternative transcription of *am778* and *am779* in tick cells and erythrocytes, respectively, similar to *ospA* and *ospC* of *Borrelia burgdorferi*.

While tools have advanced such that global transcriptional analyses are now possible for obligate intracellular organisms, it is important to keep in mind that transcription does not necessarily reflect the dynamics of protein expression. The strategy used in this study allows for a genome wide identification of differentially transcribed targets between the different conditions, however, the expression status of the encoded proteins needs to be corroborated before testing them as transmission blocking candidates. Post-transcriptional and post-translational regulation may explain discrepancies between transcript and protein data. For example, relative expression of several non-surface protein encoding genes from *A. marginale* have been studied in the bovine host versus vector environments, and previous protein expression data are not always congruent with the transcriptional data presented here. Specifically, a previous study showed that AnkC was exclusively expressed in ISE6 cells [53], while in our study transcription was decreased in ISE6 cells as compared to bovine blood. In the same study, higher expression of AnkA was observed in erythrocytes, but RNA-seq data showed no difference between transcription levels of *ankA* in the two conditions [53]. Furthermore, protein expression of AM470, AM410 and AM829 was higher in tick cells compared to blood, but only *am829* was transcribed at higher levels in tick cells [17]. Both *am470* and *am410* had equal transcription in the two conditions. Therefore, RNA-seq may be a reliable measurement of relative transcript abundance, but may not reflect protein expression.

## Conclusions

Deep sequencing technology has provided a comprehensive data set allowing global transcriptional analysis of *A. marginale* genes in the tick vector compared to bovine blood. Surface proteins were disproportionately down regulated in tick cell culture, and quantitative PCR confirmed this trend in post-acquisition tick salivary glands for a set of genes. Transcription of *A. marginale* genes in other tick samples was dissimilar to that in ISE6 cells, indicating differences in environmental conditions may result in differences in gene transcription patterns. Significant remodeling of the *A. marginale* surface in the tick vector may represent a survival strategy, a response to the lack of specific immune pressure, and evidence of specific protein functions not required in the tick. ISE6 culture is shown here to most closely mimic *A. marginale* gene transcription in the tick salivary gland environment following acquisition feeding.

## Competing interests

The authors declare that they have no competing interests.

## Authors’ contributions

GKH, SAP, GAS, KAB conceived the experiments; GKH, SAP, XC performed the experiments; GKH, SAP, XC, KAB analyzed the data; GKH, SAP and KAB wrote and edited the manuscript. All authors read and approved the final manuscript.

## Supplementary Material

Additional file 1 **Whole genome transcriptional profiling of *A. marginale* from ISE6 cell culture or blood.** The genome wide transcriptional comparison is reported. Fold changes, total gene reads, raw and normalized expression values, and p-values for Kal’s test are shown. Click here for file
